# Supplementary material for: Are carbon emissions trading and green financial instruments synergistic? -Comprehensive quantitative research based on content analysis
Source: PLoS One. 2024 Mar 7;19(3):e0298601. doi: 10.1371/journal.pone.0298601 (PMC10919601; doi:10.1371/journal.pone.0298601)
Supplement: S1 Data — (ZIP) [file pone.0298601.s001.zip › Data/地级市Z_F工作报告.zip!/║┌┴·╜¡/║┌║╙╩╨/└┤╫╘╣1⁄2╓┌║┼í╛┬φ┐╦╩2╛▌═°í┐_2007.docx]

各位代表：

现在，我代表市人民政府向大会报告工作，请予审议，并请列席会议的同志提出意见。

一、过去四年和2006年工作回顾

市三届人民代表大会以来，市政府在省委、省政府和市委的正确领导下，与全市人民一道，谋事创业，攻坚克难，为实现经济社会又好又快发展奠定了坚实基础。三次产业协调发展。2006年地区生产总值预计实现137亿元，比2002年增长47.4%，年递增10.1%，连续三年两位数增长。一产增加值实现51亿元，比2002年增长48.2%，年递增10.3%。二产增加值实现23亿元，比2002年增长37%，年递增8.1%，其中规模以上地方工业增加值实现5.6亿元，比2002年增长98.3%，年递增18.7%。三产增加值实现63亿元，比2002年增长52.2%，年递增11%。三大需求日趋旺盛。2006年城镇固定资产投资完成30.4亿元，比2002年增长52.7%，年递增11.2%。对外贸易进出口总值完成15.84亿美元，比2002年增长10倍，年递增82.1%，连续三年翻番。社会消费品零售总额实现28.8亿元，比2002年增长45.4%，年递增9.8%。发展后劲显著增强。依托区位资源优势，突出产业发育，强化项目支撑，四年累计完成投资102.4亿元，落实招商引资项目564个，到位资金43.9亿元。202国道、绥北公路建成通车，新兴基础原材料加工区、恒基水泥厂、圣泉水泥厂、海信热电厂、二门山水库、乌宋岗电站、完达山乳业、九三油脂等项目建成投产，对经济发展支撑作用开始显现。大多宝山铜矿和翠宏山铁多金属矿等大项目顺利推进，“三带十区”建设开始启动。财税收入稳步提高。2006年全市全口径财政收入实现7.01亿元，比2002年增长79.5%，年递增15.7%；一般预算收入完成3.6亿元，支出30.7亿元，比2002年分别增长56.5%和127.4%，年递增11.8%和22.8%。市直全口径财政收入实现2.2亿元，比2002年增长126.8%，年递增22.7%；一般预算收入完成1.02亿元，支出6.04亿元，比2002年分别增长70%和108.3%，年递增14.1%和20.1%。各项改革不断深化。农村综合配套改革逐步到位，2006年落实支农惠农资金2.5亿元，四年累计达到5.3亿元。国企及社保改革扎实推进，累计参加并轨企业886户，有6.9万人改变了国有职工身份，养老、医疗、生育等保险覆盖面不断扩大。粮食购销体制改革、国库集中支付改革、市县机构改革和乡镇行政编制精简工作圆满完成。人民生活逐步改善。积极扩大就业，城镇四年累计新增就业8.3万人。城镇居民人均可支配收入达到5658元，比2002年增长47.3%，年递增10.2%；农民人均纯收入达到3505元，比2002年增长46.5%，年递增10%。农村住房砖瓦化率和自来水普及率分别提高3.2和3.8个百分点。城乡居民储蓄存款余额144.2亿元，比2002年增长48.2%，年递增10.3%。社会事业全面进步。四年累计完成科技项目80项，科技贡献率不断提高。基础教育得到加强，高等教育、职业教育全面发展。市第一人民医院住院部大楼和县（市、区）疾控中心投入使用，医疗救治和疾病控制体系不断完善。人口自然增长率控制在4.56‰以下，生育符合政策率提高1.15个百分点。广播、电视覆盖率分别达到95.1%和92.3%。其他各项事业也都取得新进展。

2006年，全市上下以科学发展观为统领，解放思想，抢抓机遇，同心同德，真抓实干，较好地完成了市三届人大五次会议确定的各项任务，实现了“十一五”规划的良好开局。预计地区生产总值同比增长10.7%，城镇固定资产投资增长14%，社会消费品零售总额增长10.5%，城镇居民人均可支配收入增长12.2%，农民人均纯收入增长5.4%。一年来，主要做了以下工作。

努力促进经济增长。一产增加值同比增长6%。畜牧业、林业增加值分别完成3.9亿元和1.94亿元，增长5.8%和4.5%。种植业完成播种面积1226万亩，粮豆薯总产实现170万吨。境外土地开发达到50.1万亩，增长4倍。场县共建取得新成效，农机跨区作业达到282万亩，嫩江县与九三分局被省政府确定为共建试点单位。转移农村劳动力16.6万人，增长15.2%。组建农机合作社53个，农机装备水平明显提高。新农村建设试点顺利启动，完成投资2.6亿元。二产增加值增长14%，其中规模以上地方工业增加值增长30.5%，利税增长38.8%，是发展最快、效益最好的一年。三产增加值增长14%。接待国内外旅游者112万人次，增长14.8%，其中边境游37.4万人次，增长24.4%；实现收入14.2亿元。五大连池世界地质公园成为首批国家自然遗产，景区保护通过省人大立法。包含五大连池风景区和黑河边境游的“情系黑土地”线路在“中国十大完美旅游线路”评选中名列前茅。市区一日游正式开通。商贸餐饮等服务业日趋活跃。

不断扩大对外开放。积极组织开展中国“俄罗斯年”活动，促进了对俄经贸合作和友好往来，“两国一城”达成共识。对外贸易进出口总值同比增长171.7%，增幅列全省地市首位。口岸出入境人数达到108.5万人次，增长17.9%，首次突破百万。口岸建设服务费和互贸区非税收入实现2605万元，增长28.3%。黑龙江大桥前期工作取得新进展。布市—黑河220千伏输变电线路建设完工，输电能力可达25亿度。阿州—黑河石油输运与炼化综合体项目进展顺利，高浓度磷复合肥开发项目取得采矿权，在俄境内森林采伐40万立方米，建筑工程面积20.2万平方米。

强力推进招商引资。创新招商方式，拓展招商领域，与俄阿州、布市共同参加了“哈洽会”和“香港活动周”，成功举办了温州、苏州、杭州推介活动，推出“两国一城”国际化合作模式，引起广泛关注，荣膺2006跨国公司眼中最具投资潜力的中国城市20强之一。招商引资到位资金19.8亿元，同比增长63.6%；落实项目162项，其中投资额超亿元项目6个，超千万元项目36个。江苏梦兰、浙江合盛、大连正兴等大企业落户我市，金湾电站、海豹水泥厂、三道湾子岩金矿等一批运作多年的大项目相继开工建设。

积极增加财政收入。全市全口径财政收入同比增长20.8%，一般预算收入和支出分别增长16.5%和36.5%；市直全口径财政收入同比增长34.8%，一般预算收入和支出分别增长33%和36.5%，均有较大突破。市直医疗保险、住房公积金匹配比例达到5%，市区职工供热费补助和部门经费定额标准有所提高。加大了项目前期、招商引资、畜牧业、森林防火等投入，促进了产业发育、经济发展和安全稳定。

加快推进城乡建设。全市完成投资3.7亿元，同比增长105%。新开工建筑面积116.5万平方米，增长75%。新建住宅89.9万平方米，增长75.1%。市区完成投资2.4亿元，增长118%，重点推进了中央商业步行街改造、西部平房区改造、城市绿化、生活垃圾处理厂等项工程。新开工建筑面积45万平方米，为历史最高年份。新建改造道路21.8万平方米，新增绿地39万平方米。城管体制进一步理顺，城市环境综合整治成效显著。农村新建公路892公里，通畅率达到70.3%。生态市建设步伐加快，城市空气质量达到二级天数360天。

全面发展社会事业。完成省级以上科技项目18项。招聘重点院校毕业生109名，其中市直46名。黑河学院确立了“省市共建、以省为主”管理体制，黑河中学易地新建工程开工建设，55所农村中小学危房改造任务圆满完成，适龄儿童入学率达到99%。突发公共卫生事件处置能力进一步提高，16个乡镇卫生院建设基本完成，北安、五大连池新型农村合作医疗试点扎实推进。群众文化活动蓬勃开展，城乡举办演出700余场次。竞技体育获省级以上比赛奖牌76枚。数字有线电视前端试验平台搭建成功。双拥成果得到巩固，再次荣获省级“双拥模范城”称号。民族宗教、外事侨务、妇女儿童、老龄、残疾人及气象、人防、档案、文博图、地方史志等工作也都取得新成绩。

高度重视社会保障。强化专业技能培训，努力创造就业岗位，城镇新增就业1.76万人，登记失业率控制在4%以下。社会保障水平明显提高，职工工资和各项社会保障资金发放率均为100%。不断完善城市居民最低生活保障制度，全市城市低保人均补差标准达到84元，其中市区低保标准由150元提高到180元，并对低保户实行了供热费补贴。新建改造农村中心敬老院6所，“五保户”集中供养比例提高11个百分点。农村低保工作正式启动。

全力维护安全稳定。把安全工作放在首位，认真落实责任制，加大检查和整治力度，安全生产形势稳定好转，事故同比下降37.5%。吸取春防教训，组建了1000人的专业扑火队，实现了打好秋防翻身仗的目标。高度重视信访工作，群众反映强烈的热点难点问题得到妥善解决。强化社会治安综合治理，严厉打击各类刑事犯罪，“平安黑河”建设有了新进展。边境管理不断加强，边境秩序安全稳定。

切实加强自身建设。自觉接受市委领导，认真执行市委决策。主动接受人大法律监督和政协民主监督，全年办理人大代表建议23件，政协委员提案126件，全部办结。全面推行政务公开，扎实开展行风评议活动，群众监督机制日趋完善。制定了《黑河市人民政府重大决策规则》，决策水平进一步提高。深化行政审批制度改革，审批事项和许可项目大幅精简。认真落实廉政建设责任制，政府部门的服务意识和廉政意识明显增强。

各位代表，过去四年取得的成绩，是省委、省政府和市委正确领导的结果，是全市人民在发展中抢抓机遇、竞争中迎接挑战、艰难中顽强拼搏的结果，是人大、政协关心支持和各民主党派、工商联、无党派人士及人民团体真诚合作的结果，是人民解放军、武警官兵、公安干警支持保障和中省直单位共同努力的结果。在此，我代表市政府表示衷心感谢，并致以崇高敬意!

在总结成绩的同时，我们也清醒地看到，发展中还存在诸多困难和问题。主要表现在：农业综合生产能力不强，产业化经营水平较低，农民增收渠道不宽；支柱产业和骨干企业缺乏，资源开发步伐缓慢，工业化水平不高；第三产业层次低，进口贸易和地产品出口能力不强；财政基础薄弱，支持和保障能力有待提高；金融环境不够宽松，2006年末与年初相比，金融机构各项存款余额增长14.4%，贷款余额下降5%；一些事关群众利益的问题解决得还不够好。主观上政府部门少数同志思想不够解放，工作落实不到位，等等。对此，我们将高度重视，并采取有效措施，认真加以解决。

二、今后五年工作目标和2007年工作任务

今后五年，是我市加快发展的关键时期。我们要按照市四次党代会的部署，坚持邓小平理论和“三个代表”重要思想，全面贯彻落实科学发展观，实施“改革创新、开放升级、综合开发、强市富民”战略，扩张经济总量，增强发展活力，提高综合效益，同步协调推进经济、政治、文化、社会建设，加快建设小康黑河、和谐黑河。到2011年，地区生产总值年均增长11%，城镇固定资产投资年均增长20%，社会消费品零售总额年均增长10%，对外贸易进出口总值年均增长22%，全口径财政收入年均增长11%，城镇居民人均可支配收入年均增长10%，农民人均纯收入年均增长6%。实现这些目标，是全市人民的共同期盼，也是各级政府义不容辞的使命。

市四次党代会规划了黑河未来五年的蓝图。在落实过程中，我们必须坚持科学发展。将又好又快作为经济发展的根本要求，在加快经济增长速度的同时，注重提高质量和效益；在强化投资拉动的同时，注重刺激消费和扩大进口；在推进资源开发利用的同时，注重环境保护和节能降耗；在经济社会发展的同时，注重关注民生，提高人民群众生活水平。促进产业升级。将发展现代农业作为新农村建设的首要任务，坚持“主辅换位”，推动场县共建，壮大县域经济，提高农业效益和竞争力。将资源和能源产业开发作为提升经济总量的重中之重，强力推进“三带十区”，统筹境内外资源和能源，延长产业链，发展高载电产业，搞好进出口加工，推进工业化进程。繁荣传统服务业，加快发展现代服务业，努力提升第三产业整体水平。扩大对外开放。将扩大对俄经贸合作、增强对俄市场辐射能力作为对外开放的核心，坚持互利共赢，加快建设“两国一城”，努力提高影响力和竞争力，推动对外经贸合作向更高层次、更大范围、更宽领域发展。实施项目牵动。将项目作为拉动投资快速增长的重要支撑，规划和推进一批符合国家政策、发挥本地比较优势、便于承接发达地区资本转移，产业联结紧、带动能力强、支撑作用大的重点项目，不断蓄积发展后劲。努力开拓创新。将创新体制机制作为经济社会发展的强大动力，深化各项改革。强化开拓意识，深刻把握市情，将上级政策与黑河实际创造性地结合起来。加强对俄科技合作，增强引进消化吸收再创新能力，努力建设创新型城市。促进社会和谐。将以人为本理念作为政府工作的出发点，切实解决人民群众最关心、最直接、最现实的利益问题。做好安全稳定工作，遏制重特大森林火灾。促进社会公平正义，让人民群众共享发展成果，实现人与人、人与社会、人与自然和谐相处。

各位代表，2007年是新一届政府开局之年。我们要按照市四次党代会的总体要求，统筹兼顾，突出重点，努力实现又好又快发展。预期目标是：地区生产总值增长9%，城镇固定资产投资增长15%，社会消费品零售总额增长11%，对俄贸易进出口总值增长25%，全口径财政收入增长9%，城镇居民人均可支配收入增长10%，农民人均纯收入增长5%，万元GDP能耗降低4%。围绕上述目标，集中精力抓好以下八个方面工作。

（一）巩固和发展中国“俄罗斯年”成果，全面提高对俄经贸科技合作水平。扩大对外开放是提升我市综合竞争力的必由之路，必须充分发挥平台和通道作用，在对俄经贸科技合作战略升级上寻求新突破。努力构建“两国一城”。深入挖掘黑河与布市的对接点，实现政府互动、资源共享、市场同体、平台共用、科教同兴、文化共融，打造经贸平台、信息平台和生活平台。认真抓好两地政府会谈纪要的组织实施，完善部门间交流合作运行机制，逐步建立区域内的协调和管理体系。支持有关部门和各县（市、区）与俄对应部门和地区“结对子”，扩大在能源、矿产、农林、旅游和社会事业等领域的合作与交流。加大对外宣传力度，组织高规格研讨活动，提高“两国一城”影响力。组织参加好俄罗斯“中国年”活动。培育扶强经贸主体。落实外贸奖励政策，引进扶持壮大一批骨干经贸企业。完善对俄进出口加工区基础设施，吸引国内外企业建立生产加工基地，开展出口加工、来料加工和转口贸易。努力增加黑河口岸货运量，提高外经贸对财政的贡献率。大力优化贸易结构。积极发展边境小额贸易，稳步发展边民互市贸易，应对国家代理政策调整，规范发展一般贸易。在巩固阿州市场基础上，推动贸易领域向克拉斯诺亚尔斯克、雅库特、下卡姆斯克等俄腹地延伸。在稳定大宗进出口商品的基础上，根据国内外市场变化，加大进口企业扶持力度，积极扩大木材、电力、矿产品等资源型产品进口，进口总值增长30%；增加汽车、机电等高附加值产品出口，扩大在俄建筑工程规模，带动水泥、红砖等地工产品出口。强力推进对俄合作。继续做好境外资源调查，努力挖掘合作潜力。加快推进石油输运与炼化综合体项目，开工建设输油管道穿江段工程。继续引进俄电力，争取扩大专署供电区范围，积极参与俄远东地区电源建设和电网改造。做好扎维津斯克—逊克车陆220千伏输变电线路项目前期工作。力争高浓度磷复合肥开发项目开工建设。完成在俄境内森林采伐50万立方米，争取在布市辟建木材加工合作区。继续改善口岸环境。协调俄方完成国内审批程序，力争黑龙江公路桥尽快开工建设。完成黑河港货检现场改扩建工程。协调俄方实行双方货检现场七天工作制。争取在联检口岸互派工作人员，提高通关速度。落实好《关于保障俄罗斯公民相关权益的意见》，营造良好的治安秩序和消费环境。

（二）大力发展现代农业，扎实推进新农村建设。农业是国民经济的基础产业，也是建设小康黑河的重点和难点，必须高度重视“三农”工作，加快推进新农村建设和县域经济发展。农业增加值增长4%以上。推进北安—五大连池—嫩江现代农业产业带建设。逐步完善农产品生产基地、加工园区、物流中心和农民培训基地。启动国家大型商品粮生产基地项目，建设100万亩优质麦豆生产基地。发展优质特色高效农业，引导农民适当调减大豆面积，增加小麦、玉米、亚麻、薯类和经济作物、饲料作物面积。扩大绿色有机食品监测面积，力争达到350万亩，建立有机食品监测中心。实施现代农业高新技术示范工程和科技入户工程，推广测土配方施肥等增产新技术，提高农业科技水平。落实56个农机合作社组建任务，以机械化促进标准化。发展农村专业经济合作组织，提高农民组织化程度。积极探索土地合理流转机制和政策引导机制，促进土地集约化经营。提高场县共建层次。打破区域界线、行政级别和管理体制障碍，推进农机、农技和产业对接，实现合作层次由农业合作向工业合作延伸，合作内容由资产合作向资本合作延伸，合作领域由经济合作向社会事业领域等全方位合作延伸，促进区域经济一体化发展。整村推进达到52个，农机跨区作业面积300万亩。发挥嫩江县与九三分局共建试点带动作用，积极探索北安、五大连池与北安分局共建模式，力争实现新突破。以场县共建带动县域经济发展，全面落实省支持县域经济发展的各项政策措施，进一步扩大县级经济社会管理权限。各县（市、区）根据产业基础和区位条件确定主导产业，壮大县域经济总量。大力发展畜牧业。继续实施粮牧“主辅换位”战略，落实扶持政策，筹集专项资金。突出发展“两牛一羊”和特色养殖，提高畜牧业占整个农业的比重。做好良种引进繁育工作，强化重大疫病防治，加大先进适用技术推广力度，发展饲草饲料业，加强服务体系建设。壮大北安完达山乳业、孙吴茂田牧业、嫩江九三大洲肉业等龙头加工企业，扩大生产基地规模，探索建立企业和基地稳定、合理、紧密的利益联结机制，进一步提高经营水平。牧业增加值增长11%。发展外向型农业。扩大逊克县、爱辉区对俄蔬菜出口基地面积，力争达到10万亩。完善逊克县中俄农业科技示范园区基础设施，续建标准化节能温室，建设展示销售中心。引导农民开展对俄劳务输出，境外土地开发面积达到70万亩，增长40%。改善农民生产生活条件。加强农田基础设施建设，提高粮食综合生产能力。续建金湾等水利枢纽工程，完成象山水库除险加固，推进二道坎水库前期工作，抓好黑河城区堤防、国境界河护岸和中小河流治理。完成980公里农村公路建设任务。引导农民从事非农产业增加收入，转移农村劳动力16.8万人，劳务收入5.9亿元。加快城镇化步伐，提高小城镇辐射、带动和承载能力。继续实施农村饮水安全工程。积极发展太阳能、风能、沼气等新型清洁能源。做好20户以上已通电自然村通广播电视工作，推进乡（镇）综合文化活动中心建设。完善扶贫机制，重点抓好山区、老区扶贫。开展设立村级事业发展基金试点，坚持“一事一议”筹资筹劳制度。继续做好新农村建设试点工作。

（三）强力推进项目建设和招商引资，提高经济运行质量。工业比重低，是我市经济运行质量不高、财政实力薄弱的根本原因，必须强化投资拉动，培育优势产业集群。规模以上地方工业增加值增长18%。加快矿产资源和能源开发。加大嫩江—爱辉—逊克矿产开发产业带推进力度，探索资源勘探权和开采权的市场化。开工建设海澜热电、三吉屯年产90万吨煤矿、五大连池年产40万吨矿泉水等项目。三道湾子岩金矿、兴边煤矿、东兴煤矿投产。加快翠宏山铁多金属矿、大多宝山铜矿、海豹水泥厂等项目建设。推进新鄂水电站、乌二水电站、木耳气年产60万吨煤矿等项目前期工作。落实东安岩金矿5号矿体采矿权。重视园区载体建设。围绕原材料、能源、绿色食品、林木、医药、化工六大产业，优化政策环境和管理机制，不断增强园区的承载和集聚能力。继续推进新兴基础原材料加工区基础设施建设，建立硅基材料工程技术研究中心。北安工业园区力争进入省级工业园区行列。多宝山矿业经济区、逊克中俄合作加工区、三吉屯煤电化循环经济区争取年内有实质性进展。加快推进交通等项目。完成黑洛公路黑河至呼玛段工程，进行嫩呼公路嫩江至多宝山段和111国道嫩江段升级改造，加快嫩江至黑河公路、鹤嫩公路五大连池至嫩江段前期工作。开工建设嫩江—多宝山220千伏输变电线路，配合国家电网公司建设500千伏中俄直流背靠背交流站工程，为资源开发创造条件。抓好资源综合利用。引导热电、煤炭、建材等重点耗能行业调整结构，加快节能技术改造，发展循环经济。做好黑河热电厂、魁星啤酒公司清洁生产省级审核工作。加大新型墙体材料推广应用力度，开工建设黑河盛泰公司粉煤灰综合利用项目。推进爱辉黑金洗煤厂、北安飞龙动物制药厂等20个技改项目。加强主要行业和重点企业运行监控，抓好煤、电、油、运和重要原材料的供需衔接，保持速度和效益同步增长。提高招商引资水平。做好重大项目储备，提高项目对接度。强化招商引资意识，落实目标责任制。密切与江浙沪和香港等地的联系，拓展鲁、闽等区域，继续与阿州、布市赴国内和俄腹地联合招商，组织参加“哈洽会”和“2007中韩交流年”活动，在引进大集团方面取得新进展。做好招商后续工作，提高项目履约率。力争引进到位资金26亿元，增长30%。

（四）突出发展旅游业，拉动第三产业快速发展。第三产业是吸纳就业的主要渠道，是地方税收的主要来源，必须积极培育新兴消费热点，提升产业层次。促进旅游业提档升级。加快五大连池风景区—孙吴二战遗迹—黑河黑龙江（跨国）旅游带建设，打造休闲和体验旅游板块，重点发展火山、边境、古城、民俗、冰雪、生态和红色旅游。推进五大连池4A级景区、中俄民族风情园、爱辉历史文化旅游区等重点项目，引资建设星级宾馆，提高景区接待能力和文化品位。加快五大连池风景区机场前期工作。争取开通进京列车。搞好“完美旅游线路”宣传推介，提升国内外知名度。继续争取恢复异地办照，激活出境旅游市场。接待国内外旅游者125万人次，增长12%；旅游总收入16亿元，增长14%。大力发展物流业。发展试车经济，筹建黑河汽车出口展示中心，建设寒带试车、国际车展、汽车出口“三位一体”国际试车基地。加强中心市场和专业批发市场建设，大黑河岛互市贸易广场和嫩江建材、农机大市场投入运营，北安物流中心开工建设。继续推进“万村千乡”市场工程，建设以农产品、轻工、建材等商品集散为重点，连接内地和城乡，辐射境外市场的区域物流体系。提升传统服务业。进一步发挥互市贸易区载体功能，提高商贸流通、餐饮娱乐服务质量。适应俄游客需求，在公共服务窗口和商场、宾馆、饭店、娱乐场所悬挂双语牌匾，实行双语标价，印制中俄文市区交通地图和商务指南。加强市场监控，打击假冒伪劣，整顿流通秩序，保障食品、药品和餐饮卫生安全。培育新型服务业。支持法律、会计、金融、保险、信息、咨询、广告等服务业发展，提高中介执业水平。利用独特的外景条件，发展影视服务业。加强社区建设，鼓励社会各方面力量，兴办以零售连锁、医疗卫生、家政服务为重点的社区服务业，满足日益增长的社会需求。

（五）坚持改革创新，增强经济社会发展动力。破解体制性、机制性矛盾是加快我市经济社会发展的重要途径，必须坚持市场经济方向，积极稳妥推进重点领域和关键环节的改革。启动事业单位改革。市县两级同步推进，在科学分类的基础上，制定方案，整合资源，出台相关配套政策，逐步建立和完善公开、平等、竞争、择优的选人用人机制和激励分配机制，增强事业单位发展活力，不断提高公益服务质量。推进国有林权制度改革。抓好爱辉区试点，年内完成75万亩国有商品林地流转任务。及时总结试点经验，适时在全市范围推开。继续引资开展合作造林，活化宜林荒山荒地使用权。大力发展林产经济，在发挥生态效益的同时，提高经济效益。深化国企改革。完成西沟水电公司股权置换。深化黑河地方铁路（集团）公司和黑河热电厂等骨干企业内部改革，创新管理机制。实施好北安庆华厂厂办大集体并轨试点工作。指导151煤矿、东方红煤矿等新改制企业，完善法人治理结构，健全现代企业制度。在重点抓好上述改革的同时，继续推进农村综合改革和投资、财政体制等改革。大力支持民营经济。坚持“非禁即入、平等对待”，支持民营资本进入基础设施、社会公用事业等领域，参与国企改革和重组。鼓励和引导民营企业以资本联合等方式，扩大规模，提升层次，增强竞争力。鼓励科技创新。培育科技创新载体，扶持高新技术企业，抓好12项省级以上重点科技项目实施，促进科技成果转化与应用。搞好国内科技共建，加强与科研院所和重点院校的联系。巩固对俄科技合作成果，注重俄先进技术的引进消化吸收和再创新。加强人才队伍建设，做好培养和引智工作，发挥学科带头人作用，继续招聘储备重点本科院校毕业生，为科技创新提供人才保障。

（六）提升城市品位，建设独具特色的现代化口岸城市。城市环境与百姓生活密切相关，群众关注度高，必须加强城市规划建设管理，努力改善人居环境。科学把握城市定位。聘请国家级专家组，对市区建成区进行诊断，对今后城市建设进行论证。突出沿边临水的城市特点，重点明确市区沿江风光带、大黑河岛、黑龙江大桥桥头区和西部平房区的基本功能、建筑风格和空间布局等，做到市、区协调统一。强化规划地位和作用。高标准组织编制城市各类专业规划、分区规划和详细规划，新增详细规划面积100公顷。继续实行规划和土地集体审批、集中审定制度，加大规划行政监管力度，坚决维护城市规划的权威性和严肃性。推进建设项目规范化。采取政府引导和市场运作相结合的方式，制定相应政策，加快市区西部平房区改造。完成海南路、中央商业步行街等续建工程，新开工建设中央街东段环城路至黑河学院拓宽和龙滨路、龙源路升级改造工程。指导黑河学院推进文体设施建设，实现共建共享。建设项目做到早确定、早施工、早受益，讲程序、讲规范、讲质量、讲效益。实施精细化管理。认真总结城市管理经验，围绕保洁、路灯、绿化、广告牌匾等具体管理内容，体现以人为本，出台管理办法，建立和完善管理机制。推进物业管理产业化，逐步扩大覆盖面。加快生态市建设，强化源头治理，严把建设项目环保审批关。以创建“无烟城市”为载体，集中整治城市烟尘污染源。解决新兴基础原材料加工区环境污染问题。开展“市容市貌改善年”活动。巩固扩大“城市道路年”和“城市绿化年”成果，进一步硬化、绿化、亮化、美化和净化。除抓好重点基础设施项目外，硬化巷道20条，改造住宅小区15个，规划建设停车场。通江路及公共建筑物进行灯饰亮化，老城区主要街路建筑进行立面修饰，丰富黑龙江公园文化内涵，提高城市文化品位。继续开展以“治脏、治乱、治荒”为重点的环境整治。各县（市）根据本地实际，加快道路、给排水等基础设施建设，不断改善城乡面貌。

（七）做好财税金融工作，为经济社会发展提供有力支撑。财税金融工作既关系到经济发展，又关系到社会稳定，必须坚持开源节流，改进服务，努力实现财税金融良性运转。坚持依法治税。加大税源监控力度，做好税收征管工作。继续完善税源动态管理机制，实行以票管税。健全非税收入管理机制，严格执行“收支两条线”管理。加大财税稽查力度，严厉打击偷逃骗税，严肃查处违规行为，确保应收尽收。保证重点支出。不断优化支出结构，确保调整职工工资和津贴政策足额兑现，确保各项社会保障资金及时足额发放。增加对新农村、畜牧业、项目前期、招商引资和科教文卫领域的投入，提高财政公共保障能力。搞好重点支出和专项资金监督检查，实行财政支出绩效评价。抓好财源建设。在巩固现有财源基础上，大力培植新兴财源、后续财源，特别是支柱财源。已经立项的财源项目，积极拓宽融资渠道，确保如期开工、投产达效。搞好项目筛选、储备和立项，争取更多项目纳入省重点财源建设扶持范围。积极向上争取。深入研究和及时掌握上级政策及专项资金渠道，根据黑河实际，重点围绕新农村建设、资源开发、经贸旅游以及教育、环保、森林防火等方面，积极争取政策、资金、项目、贷款、装备和指标，提高财政支持经济社会发展的能力。改善金融服务。进一步优化金融环境，协调金融机构扩大信贷规模。充分发挥市担保公司作用，积极寻求与金融机构合作，着力搭建银企桥梁，努力缓解企业贷款难的问题。完成6个县（市、区）农村信用合作社统一法人制改革，提高金融支持经济发展的能力。

（八）切实关注民生，努力建设和谐黑河。社会和谐既是经济发展的目的，又是经济发展的保障，必须从解决人民群众最关心、最直接、最现实的利益问题入手，促进经济社会协调发展。抓好就业和社会保障。落实扶持政策，积极开展培训，拓宽就业渠道，城镇登记失业率控制在4.5%以内。黑河对俄劳务输出实训基地年内完工。切实维护劳动者合法权益。加强基金征缴，扩大城镇养老、医疗、工伤和生育保险覆盖面。进一步完善城乡低保和社会救助制度，做好农村养老保险工作，逐步建立覆盖城乡的社会保障体系。继续开展城市“一帮一”扶贫解困和“献爱心、送温暖”活动。落实《黑河市城区最低收入家庭廉租住房管理办法》，改善城镇困难群众居住条件。优先发展教育事业。支持黑河学院做好国家本科院校教学水平评估工作，加快学院基础设施建设，加强与俄高校交流，推动中俄教育合作向更深层次拓展。高质量完成黑河中学易地新建工程，保证年内投入使用。扩大高中招生规模，加强教师队伍建设，提高教学水平。巩固扩大“两基”成果，加大基础教育课改力度，扎实推进素质教育。严格执行一费制，控制学校收费标准。加强农村义务教育，落实贫困家庭学生免杂费、课本费和补助寄宿学生生活费政策，建立农村中小学危房改造长效机制。发展职业教育，调整专业结构，做好五大连池、逊克职教中心晋级申报工作，培养实用人才。积极推进卫生事业。坚持公共医疗卫生的公益性质，加快县乡村三级农村医疗卫生服务网络建设。全面推行新型农村合作医疗制度，范围扩大到6个县（市、区）。逐步完善社区卫生服务功能，不断扩大覆盖面。加强公共卫生体系建设，提高突发公共卫生事件应急处置能力、重大传染病预防控制能力和医疗救治能力。加强中俄卫生交流合作，发展特色专科，提高医疗服务水平。开工建设市第一人民医院门诊部大楼。巩固和提高人口与计划生育工作水平，生育符合政策率达到95%。大力发展文体事业。加快和谐文化建设，打造“边疆文化名城”。继续深化中俄文化交流合作，建设交流平台。开工建设市青少年活动中心和广电中心。深入开展“城市之光”和“金色田野”主题文化活动，丰富群众文化生活。发展竞技体育，培养优秀体育人才。健全群众性体育基层组织网络，推动全民健身运动深入开展。统筹发展其他社会事业。加大政府投入，引导社会力量共同兴办福利事业。支持老龄工作，关心残疾人事业。进一步做好民族宗教、外事侨务、妇女儿童以及气象、人防、档案、文博图、地方史志等工作。加强国防动员和民兵预备役建设，妥善安置军转干部和复员军人。做好拥军优属和拥政爱民工作，争创全国“双拥模范城”。全力维护安全稳定。以高度责任心抓好森林防火工作，进一步落实领导责任，提高全民森防意识，加强防火基础建设，打好森林防火的主动仗。加强煤矿安全监管，做好小煤矿关闭整顿工作。继续抓好交通、易燃易爆品、公共场所等方面的专项整治，坚决遏制重特大事故发生。加强应急机构、应急队伍和救援体系建设，确保应急救援快速有效。认真贯彻《信访工作条例》，不断完善矛盾纠纷排查调处机制，妥善处理信访突出问题和群体性事件。坚持严打方针，加强社会治安综合治理，严厉打击各类刑事犯罪，建设“平安黑河”。加强边防基础设施建设，维护边境安全和谐稳定。

三、加强政府自身建设，进一步提高行政效能

面对历史的重任，群众的期盼，政府必须努力提高工作水平，开创工作新局面。坚持科学民主决策。主动接受市委领导，坚决贯彻市委决策。认真落实市人大及其常委会决议，自觉接受人大法律监督和政协民主监督，认真办理人大代表议案、建议和政协委员提案，听取各民主党派、工商联和各人民团体的意见。认真执行政府重大决策规则，重大问题常务会议集体讨论决定。建立健全专业性较强的重大事项决策专家论证、技术咨询制度，与群众利益密切相关的重大事项决策公示、听证制度，善于利用“外脑”，提高决策水平。全面推行政务公开，广泛接受社会监督。加快政府职能转变。在加强经济调节和市场监管的同时，更加注重履行社会管理和公共服务职能。认真贯彻《全面推进依法行政实施纲要》，严格执行行政执法责任制和行政过错责任追究制，做好行政复议工作，依法管理社会事务。深化行政管理体制和行政审批制度改革，探索建立行政服务中心，减少审批环节，提高行政效率和服务水平。提高推进落实能力。认真贯彻实施《公务员法》，严格执行《黑河市国家公务员公共服务行为问责暂行规定》，加强管理和培训，不断提高公务员队伍素质。进一步增强责任心和紧迫感，始终保持脚踏实地的务实作风、自我加压的主动意识和争先创优的进取精神。健全工作推进机制，完善目标考核体系，强化跟踪督办检查，狠抓各项决策落实，全面提高政府工作绩效。切实加强廉政建设。坚决贯彻反腐倡廉各项规定，认真落实党风廉政建设责任制，在重大决策、重点项目和大额资金使用等方面健全制度，完善权力运行监督机制。发挥行政监察和审计机关职能作用，严肃查办违法违纪案件。继续开展行风评议活动，严格治理乱收费、乱罚款、乱摊派行为，认真纠正部门和行业不正之风。努力建设诚信政府。在政府部门和公务员中树立诚信理念和责任理念，忠实履行对人民承担的职责，强化工作时效性，言必行，行必果，以实际行动取信于民，提高政府执行力和公信力。注重保持政策的连续性和稳定性，建立政府诚信问责制度，严处行政失信行为。创造良好信用环境，推动“诚信黑河”建设。

各位代表，新的目标令人鼓舞，新的形势催人奋进，新一届政府将承担更为艰巨而光荣的历史使命。让我们在市委的正确领导下，紧紧依靠全市人民，进一步解放思想，开拓创新，奋力拼搏，真抓实干，为全面建设小康黑河、和谐黑河而努力奋斗！
